# Associations between Active Travel to Work and Overweight, Hypertension, and Diabetes in India: A Cross-Sectional Study

**DOI:** 10.1371/journal.pmed.1001459

**Published:** 2013-06-11

**Authors:** Christopher Millett, Sutapa Agrawal, Ruth Sullivan, Mario Vaz, Anura Kurpad, A. V. Bharathi, Dorairaj Prabhakaran, Kolli Srinath Reddy, Sanjay Kinra, George Davey Smith, Shah Ebrahim, for the Indian Migration Study group

**Affiliations:** 1 School of Public Health, Imperial College, London, United Kingdom; 2 South Asia Network for Chronic Disease, Public Health Foundation of India, New Delhi, India; 3 Department of Non-communicable Disease Epidemiology, London School of Hygiene & Tropical Medicine, London, United Kingdom; 4 St. John's Research Institute, Bangalore, India; 5 Indira Gandhi National Open University, Bangalore, India; 6 Centre for Chronic Disease Control, New Delhi, India; 7 Public Health Foundation of India, New Delhi, India; 8 School of Social and Community Medicine, University of Bristol, Bristol, United Kingdom; Johns Hopkins Bloomberg School of Public Health, United States of America

## Abstract

**Background:**

Increasing active travel (walking, bicycling, and public transport) is promoted as a key strategy to increase physical activity and reduce the growing burden of noncommunicable diseases (NCDs) globally. Little is known about patterns of active travel or associated cardiovascular health benefits in low- and middle-income countries. This study examines mode and duration of travel to work in rural and urban India and associations between active travel and overweight, hypertension, and diabetes.

**Methods and Findings:**

Cross-sectional study of 3,902 participants (1,366 rural, 2,536 urban) in the Indian Migration Study. Associations between mode and duration of active travel and cardiovascular risk factors were assessed using random-effect logistic regression models adjusting for age, sex, caste, standard of living, occupation, factory location, leisure time physical activity, daily fat intake, smoking status, and alcohol use. Rural dwellers were significantly more likely to bicycle (68.3% versus 15.9%; *p*<0.001) to work than urban dwellers. The prevalence of overweight or obesity was 50.0%, 37.6%, 24.2%, 24.9%; hypertension was 17.7%, 11.8%, 6.5%, 9.8%; and diabetes was 10.8%, 7.4%, 3.8%, 7.3% in participants who travelled to work by private transport, public transport, bicycling, and walking, respectively. In the adjusted analysis, those walking (adjusted risk ratio [ARR] 0.72; 95% CI 0.58–0.88) or bicycling to work (ARR 0.66; 95% CI 0.55–0.77) were significantly less likely to be overweight or obese than those travelling by private transport. Those bicycling to work were significantly less likely to have hypertension (ARR 0.51; 95% CI 0.36–0.71) or diabetes (ARR 0.65; 95% CI 0.44–0.95). There was evidence of a dose-response relationship between duration of bicycling to work and being overweight, having hypertension or diabetes. The main limitation of the study is the cross-sectional design, which limits causal inference for the associations found.

**Conclusions:**

Walking and bicycling to work was associated with reduced cardiovascular risk in the Indian population. Efforts to increase active travel in urban areas and halt declines in rural areas should be integral to strategies to maintain healthy weight and prevent NCDs in India.

*Please see later in the article for the Editors' Summary*

## Abstract 2

Using data from the Indian Migration Study, Christopher Millett and colleagues examine the associations between active travel to work and overweight, hypertension, and diabetes.

*Please see later in the article for the Editors' Summary*

## Editors' Summary

**Background:**

Noncommunicable diseases (NCDs) and obesity (excessive body fat) are major threats to global health. Every year, more than 36 million people (including 29 million in LMICs) die from NCDs—nearly two-thirds of the world's annual deaths. Cardiovascular diseases (conditions that affect the heart and the circulation), diabetes, cancer, and respiratory diseases are responsible for most NCD-related deaths. Obesity is a risk factor for all these NCDs and the global prevalence of obesity (the proportion of the world's population that is obese) has nearly doubled since 1980. In 2008, 35% of adults were overweight and 11% were obese. One reason for the growing burden of both obesity and NCDs is increasing physical inactivity. Regular physical activity helps to maintain a healthy body weight and to prevent or delay the onset of NCDs. For an adult, 30 minutes of moderate physical activity—walking briskly or cycling, for example—five times a week is sufficient to promote and maintain health. But the daily lives of people in both developed and developing countries are becoming increasingly sedentary and, nowadays, at least 60% of the world's population does not do even this modest amount of exercise.

**Why Was This Study Done?:**

Strategies to increase physical activity levels often promote active travel (walking, cycling, and using public transport). The positive impact of active travel on physical activity levels and cardiovascular health is well established in high-income countries, but little is known about the patterns of active travel or the health benefits associated with active travel in poorer countries. In this cross-sectional study (an investigation that measures population characteristics at a single time point), the researchers examine the mode and duration of travel to work in rural and urban India and associations between active travel and overweight/obesity, hypertension (high blood pressure, a risk factor for cardiovascular disease), and diabetes. In India, a lower middle-income country, the prevalence of overweight and NCDs is projected to increase rapidly over the next two decades. Moreover, rapid unplanned urbanization and a large increase in registered motor vehicles has resulted in inadequate development of the public transport infrastructure and hazardous conditions for walking and cycling in most Indian towns and cities.

**What Did the Researchers Do and Find?:**

For their study, researchers analyzed physical activity and health data collected from participants in the Indian Migration Study, which examined the association between migration from rural to urban areas and obesity and diabetes risk. People living in rural areas were more likely to cycle to work than people living in towns and cities (68.3% versus 15.9%). Among people who travelled to work by private transport, public transport, walking, and cycling, the prevalence of overweight or obesity was 50.0%, 37.6%, 24.9%, and 24.2%, respectively. Similar patterns were seen for the prevalence of hypertension and diabetes. After adjustment for factors that affect the risk of obesity, hypertension, and diabetes (for example, daily fat intake and leisure time physical activity), people walking or cycling to work were less likely to be overweight or obese than those travelling by public transport, and those cycling to walk were less likely to have hypertension or diabetes. Finally, people with long cycle rides to work had a lower risk of being overweight or having hypertension or diabetes than people with short cycle rides.

**What Do These Findings Mean?:**

These findings suggest that, as in high-income settings, walking and cycling to work are associated with a reduced risk of cardiovascular disease in India. Because this was a cross-sectional study, these findings do not prove that active travel reduces the risk of cardiovascular disease—people who cycle to work may share other unknown characteristics that are actually responsible for their reduced risk of cardiovascular disease. Moreover, this study did not consider non-cardiovascular outcomes associated with active travel that might affect health such as increased exposure to air pollution. Nevertheless, these findings suggest that programs designed to maintain healthy weight and prevent NCDs in India should endeavor to increase active travel in urban areas and to halt declines in rural areas by, for example, increasing investment in public transport and improving the safety and convenience of walking and cycling routes in urban areas.

**Additional Information:**

Please access these Web sites via the online version of this summary at http://dx.doi.org/10.1371/journal.pmed.1001459.

- This study is further discussed in a *PLOS Medicine* Perspective by Kavi Bhalla
- The US Centers for Disease Control and Prevention provides information on all aspects of healthy living, on chronic diseases and health promotion, on overweight and obesity and on non-communicable diseases around the world; its Physical Activity for Everyone web pages include guidelines, instructional videos and personal success stories (some information in English and Spanish)
- The World Health Organization provides information about physical activity and health, about obesity, and about non-communicable diseases (in several languages); its 2010 Global Recommendations on Physical Activity for Health are available in several languages; its Global Noncommunicable Disease Network (NCDnet) aims to help low- and middle- income countries reduce NCD-related illnesses and death through implementation of the 20082013 Action Plan for the Global Strategy for the Prevention and Control of Noncommunicable Diseases (also available in French); Face to face with chronic diseases is a selection of personal stories from around the world about dealing with NCDs
- The American Heart Association provides information on many important risk factors for non-communicable diseases and provides tips for becoming more active
- Information about the Indian Migration Study is available

## Introduction

Active travel (walking, bicycling, and use of public transport) is increasingly being promoted as an integral component of strategies to increase physical activity levels and address the growing burden of obesity and non-communicable diseases (NCDs) globally [1],[2]. The Action Plan of the World Health Organization's (WHO's) Global Strategy for the Prevention and Control of NCDs urges member states to “introduce transport policies that promote active and safe methods of travelling to and from schools and workplaces” and to “ensure that physical environments support safe active commuting.” [3]. While a number of high-income countries, most notably the Netherlands, Denmark, and Germany, have implemented effective policies to increase active travel [4], such efforts remain largely undeveloped in low- and middle-income countries (LMICs) such as India, where the bulk of the NCD burden falls.

Efforts to increase active travel in India face a number of powerful countervailing influences, including rapid, unplanned urbanisation and substantial growth in motor vehicle ownership. The percentage of the Indian population living in urban areas increased from 23% in 1980 to 31% in 2010 and is projected to exceed 50% by 2050. There was a 38-fold increase (3 to 115 million) in the number of registered motor vehicles in the country between 1981 and 2009 [5]. Successive governments have prioritised investment in road infrastructure since the National Highway Act in 1995 and planning for urban growth at the local level has generally been weak and haphazard [6]. In combination, these factors have resulted in inadequate development of the public transport infrastructure and hazardous conditions for walking and bicycling in most Indian cities and towns.

The positive impacts of active travel on physical activity levels and cardiovascular health are well established in high income countries [7],[8]. For example, a US study found that users of public transport, walk for an average of 19 min as part of their daily commute and that 29% of public transport users achieve recommended levels of daily physical activity from this travel alone [9]. However, there has been little research examining patterns of active travel and the associated health benefits in India and other LMICs. Further, there is sparse information on how patterns of active travel differ in rural and urban India. This is an important knowledge gap given that the prevalence of overweight, diabetes, and cardiovascular disease (CVD) is substantially higher in urban India [10]–[12]. This study has two aims: (1) to characterise modes of active travel to work in urban and rural populations in India; (2) to examine associations between modes of active travel (walking, bicycling, public transport) to work and overweight and obesity, hypertension, and diabetes in India.

## Methods

### Study Design and Respondents

This study used data from the Indian Migration Study (IMS) conducted during 2005–2007. The design and sampling methodology of the IMS has been described previously [10],[13]. Briefly, the IMS is a cross-sectional sib-pair study, part of a larger cardiovascular risk factor surveillance system [14]. The IMS was carried out in factory settings in four cities from northern (Lucknow, Hindustan Aeronautics Ltd), central (Nagpur, Indorama Synthetics Ltd), and southern India (Hyderabad, Bharat Heavy Electricals Ltd; and Bangalore, Hindustan Machine Tools Ltd). Information on rural-to-urban migration was solicited from factory workers and their co-resident spouses. Factory workers and their co-resident spouses who had migrated from rural to urban areas, along with a 25% random sample of urban non-migrants and their co-resident spouses, were asked to participate in the study. Each migrant participant was asked to identify a non-migrant sibling residing in a rural area, preferably of the same gender and close to them in age, who was then also invited to participate in the study. In a small number of cases where no rural sibling was available (<5%), a cousin or a close friend from the same village was invited. There were no other exclusion criteria at this recruitment stage. This convenience sampling strategy resulted in rural dwelling siblings being drawn from anywhere in the country (18 of the 29 states), reflecting the migration patterns of the factory workers and their spouses. A substantial proportion came from the four large states in which the factories were based (Uttar Pradesh, Maharashtra, Andhra Pradesh, and Karnataka). The urban participants were also asked to identify a non-migrant, urban dwelling sibling for inclusion in the study.

Of the 7,594 migrant and non-migrant factory workers and their co-resident spouses identified as being eligible for the India Migration Study, 7,102 (94%) agreed in principle to complete the clinical examination with their sibling, of whom 3,537 (50%) sib-pairs eventually participated by the close of field work. The final IMS sample was 7,067 respondents as seven respondents did not complete the clinical examination. Our analysis is based on 3,902 respondents aged ≥18 y who reported being in the workforce. Exclusions included respondents who reported being unemployed/did housework (2,508), had no information on their mode of transport to work (308), or had no information on their migration status (349). Ethics committee approval for the IMS was obtained from the All India Institute of Medical Sciences Ethics Committee, reference number A-60/4/8/2004.

### Data Collection and Measurements

An interviewer-administered questionnaire was used to collect socio-demographic, physical activity, and health data from respondents. Trained personnel took anthropometric measures of height and weight from all participants during a clinic visit. Height was measured to the nearest 0.1 cm using a portable stadiometer with a base plate (Leicester height measure, Chasmore Ltd.). Weight was measured twice, to the nearest 0.1 kg using a digital scale (Model PS16), with participants removing their shoes and wearing light clothing. Blood pressure was measured twice using an Omron M5-I automatic machine in sitting position using the right upper arm and an appropriate sized cuff after a period of 5-min rest. The average of these two measures taken was used for these analyses. Participants were asked to attend the clinic visit fasting and the time of last meal was recorded. Glucose was measured on the day of blood sample collection in local laboratories at each site with the GOD-PAP method using RANDOX kits. The quality of local assays was checked with regular external standards and internal duplicate assays and monitored by a reference laboratory at the All India Institute of Medical Sciences. Daily fat intake was assessed from a validated, interviewer-administered semi-quantitative food frequency questionnaire which is described elsewhere [15]. The questionnaire assessed frequency of intake (daily, weekly, monthly, yearly) of 184 commonly consumed food items. Information collected in the food frequency questionnaire was converted into average daily consumption of nutrient and food groups, using nutrient databases that were developed for the study. A validated, interviewer-administered questionnaire was used to assess physical activity in the past month across multiple domains including discretionary leisure time, household chores, work, sleep, sedentary activities, and other common daily activities in the IMS [16]. Respondents were asked whether they undertook sports, games, or other physical activities such as walking for leisure. Those responding affirmatively were asked about the frequency (daily; 5–6 times/week; 2–4 times/week; once a week, 2–3 times/month; once a month) and the average duration in minutes of each leisure activity undertaken. Metabolic equivalent tasks (METs) were estimated as the ratio of resting metabolic rate where 1 MET is equivalent to the energy expenditure value of sitting quietly using an established method [16]. Activity data, including for leisure time physical activity, were summarised as MET hours per day. All protocols and equipment were pilot tested prior to the study commencing. Fieldworkers at the four study sites underwent joint training sessions and standardisation at the outset and subsequently every 6 mo. Anthropometric instruments were calibrated at the start of each clinic session.

### Predictor Variable

#### Active Travel To Work

Information on mode and duration of travel to work was gathered from respondents as part of the questionnaire on physical activity [16]. Mode of transport to work was categorized as private transport (car and motorcycles), public transport (three wheeler, bus, and train), walking and bicycling. Respondents were asked to estimate the duration of travel to their workplace in minutes. We doubled this to obtain the total amount of daily travel to and from the workplace. We categorized respondents according to whether their daily active travel (to and from work) was 30 min or greater, reflecting international guidelines on recommended physical activity levels [1].

### Outcome Variables

Overweight was defined using two cut points; body mass index (BMI)≥23 kg/m^2^ (suitable for Asian populations [17]) and BMI≥25 kg/m^2^. Obesity was defined as BMI≥30 kg/m^2^. Hypertension was defined as a report of a doctor diagnosis of hypertension. Undiagnosed hypertension was defined as a systolic BP≥140 mm Hg or a diastolic BP≥90 mm Hg in the absence of a doctor diagnosis. Diabetes was defined as a report of a doctor diagnosis of diabetes. Undiagnosed diabetes was defined by WHO fasting plasma glucose criterion of >7.0 mmol/l in the absence of a doctor diagnosis of diabetes [18]. Homeostasis model assessment (HOMA) scores to estimate insulin resistance were calculated from fasting blood glucose and serum insulin levels using a standard formula of plasma glucose (mol/l)×plasma insulin (mU/l)/22.5), on the basis of the original approach [19]. HOMA has been validated by comparison with biochemical markers of insulin resistance in healthy Indian people, yielding moderate correlations [20]. We have divided the HOMA scores into tertiles and created a binary variable (high versus medium/low). Our rationale for using both doctor diagnosed and undiagnosed hypertension and diabetes as outcome measures was to address the possibility of reverse causality, i.e., individuals with diagnosed diabetes and hypertension may receive physician advice to increase physical activity. The number of participants with missing data for our main outcomes was negligible (<10 respondents for each variable). Full details on data completeness in the study can be found elsewhere [10].

### Covariates

Covariates in our analysis included age, sex, caste/tribe status, standard of living index (SLI), occupation, factory location, leisure time physical activity (MET hours/day), daily fat intake (grams per day), current alcohol intake (yes/no), and smoking status (current smoker/non smoker). “Scheduled castes” and “scheduled tribes” are identified by the Government of India as socially and economically backward and needing protection from social injustice and exploitation. “Other backward class” is a diverse collection of intermediate castes that were considered low in the traditional caste hierarchy but are clearly above scheduled castes. “General” is thus a default residual group that enjoys higher status in the caste hierarchy. The SLI is based on following assets in the household: quality of house; toilet facilities; source of lighting and drinking water; possession of clock, radio, television, bicycle, motorcycle, car, tractor, refrigerator, and telephone. A score of less than 20 indicates low SLI, 21–26 indicates medium SLI, and 27 and above indicates high SLI. Occupation was categorized as manual and non-manual.

### Statistical Analysis

As the study is based on factory workers, their spouses, and a sibling of each factory worker and spouse these data cannot be treated as coming from independent individuals and the data structure must be accounted for in the statistical analysis [10]. A general multilevel random effects model framework that can accommodate this data structure was used [21]. In the multilevel model the between-pair variation is specified explicitly and included in the model.

Standard descriptive analysis was done using chi square tests. Association between mode of transport and duration of travel was assessed using random-effect logistic regression models after adjusting for the sib-pair design, factory location, and potential confounders. Separate models were run with BMI and MET hours/day as additional covariates. Comparisons were made between mode of transport and duration of travel using the logistic regression with a pair-specific random effect to estimate the within-pair comparisons for all the binary outcomes. Odds ratios for associations between mode of transport and outcomes were calculated and adjustments made for age, sex, caste, standard of living, occupation, factory location, leisure time physical activity, daily fat intake, smoking status, and alcohol use as these may confound the associations. We then corrected for possible overestimation of odds ratios for common outcomes by adjusting to approximate risk ratios using an established method [22]. All statistical analyses were conducted using STATA software version 10 (StataCorp. 2009. Stata Statistical Software: Release 10. StataCorp LP).

## Results

Of the 3,902 eligible participants, 1,366 were rural dwellers and 2,536 were urban dwellers (Table 1). There were no major differences between migrant and non-migrant urban dwellers so they were combined into a single group. The mean age of respondents was 42.2 y (standard deviation [SD] 9.6) and 87.2% were men. A significantly higher percentage of rural dwellers were employed in manual occupations than urban dwellers (75.1%, 51.9%, respectively; *p*<0.001). Rural dwellers were significantly more likely to belong to lower caste groups and be in the lowest SLI group than (69.8%, 16.7%; *p*<0.001). Rural dwellers were significantly less likely to be overweight (19.1%, 44.5%; *p*<0.001) and significantly less likely to have doctor diagnosed hypertension (5.1%, 15.3%; *p*<0.001) or doctor diagnosed diabetes (2.8%, 9.7%; *p*<0.001) than urban dwellers.

**Table 1:** Characteristics of the study sample.

Characteristics	Rural Dwellers *n* = 1,366	Urban Dwellers *n* = 2,536	Total *n* = 3,902	χ^2^ *p*-value
	*n* [%]	*n* [%]	*n* [%]	
**Mean age [±SD]**	40.3 [±10.5]	43.2 [±8.8]	42.2 [±9.6]	
**Sex (% men)**	1,228 [89.9]	2,174 [85.7]	3,402 [87.2]	<0.001
**Occupation**				
Manual jobs	1,026 [75.1]	1,316 [51.9]	2,342 [60.0]	<0.001
Non-manual jobs	340 [24.9]	1,220 [48.1]	1,560 [40.0]	
**Caste/tribe status**				
Scheduled castes	253 [18.5]	383 [15.1]	636 [16.3]	<0.001
Scheduled tribes	94 [6.9]	96 [3.8]	190 [4.9]	
Other backward class	473 [34.7]	810 [32.0]	1,283 [32.9]	
General	545 [39.9]	1,246 [49.2]	1,791 [45.9]	
**Standard of living**				
Low	953 [69.8]	424 [16.7]	1,377 [35.3]	<0.001
Medium	294 [21.5]	1,141 [45.0]	1,435 [36.8]	
High	119 [8.7]	971 [38.3]	1,090 [27.9]	
**Smoking status**				
Non smoker	1,100 [80.5]	2,165 [85.4]	3,265 [83.7]	<0.001
Current smoker	266 [19.5]	371 [14.6]	637 [16.3]	
**Current alcohol intake**				
No	1,059 [77.5]	1,960 [77.3]	3,019 [77.4]	0.87
Yes	307 [22.5]	576 [22.7]	883 [22.6]	
**Mean dietary fat intake [±SD]** a	77.3 [±37.2]	93.5 [±37.0]	87.8 [±37.9]	
**Mean leisure time PA [±SD]** b	1.1 [±2.2]	1.6 [±2.2]	1.4 [±2.2]	
**Outcome measures**				
BMI≥23 kg/m^2^	453 [33.2]	1,674 [66.0]	2,127 [54.5]	<0.001
BMI≥25 kg/m^2^	261 [19.1]	1,127 [44.5]	1,388 [35.6]	<0.001
BMI≥30 kg/m^2^	40 [2.9]	212 [8.4]	252 [6.5]	<0.001
Doctor reported hypertension	69 [5.1]	388 [15.3]	457 [11.7]	<0.001
Undiagnosed hypertension	115 [8.4]	261 [10.3]	376 [9.6]	0.16
Doctor reported diabetes	38 [2.8]	245 [9.7]	283 [7.3]	<0.001
Undiagnosed diabetes	31 [2.3]	91 [4.0]	122 [3.4]	0.02
HOMA score^c^				
High HOMA	332 [25.7]	1,353 [62.3]	2,311 [66.8]	<0.001
Low/medium HOMA	958 [74.3]	818 [37.7]	1,150 [33.2]	

*p*-values indicate whether sample characteristics differ significantly in rural and urban respondents.

a Mean dietary fat intake (grams per day).

b Mean leisure time physical activity (MET hours per day).

c HOMA score excludes doctor diagnosed diabetes cases and cases where fasting blood glucose > = 7 mmol/l.

doi:10.1371/journal.pmed.1001459.t001

### Mode and Duration of Travel to Work

Bicycling was the commonest mode of travel to work among the rural dwellers (68.3%), followed by private vehicle (12.5%), walking (11.9%), and public transport (7.3%) (Table 2). The commonest mode of travel to work in urban dwellers (44.5%) was private vehicle. 27.1% of urban dwellers used public transport to travel to work, 15.9% bicycled to work, and 12.5% walked to work.

**Table 2:** Percent using different travel modes to work and duration of travel in urban and rural dwellers.

Location	Private Transport		Public Transport		Bicycle		Walking	
	Percent [*n*]	Mean Duration [95% CI]	Percent [*n*]	Mean Duration [95% CI]	Percent [*n*]	Mean Duration [95% CI]	Percent [*n*]	Mean Duration [95% CI]
Rural	12.5 [171]	39.3 [33.1–45.6]	7.3 [100]	88.0 [75.4–100.5]	68.3 [933]	38.6 [36.4–40.8]	11.9 [162]	48.0 [42.0–54.1]
Urban	44.5 [1,129]	26.2 [24.7–27.6]	27.1 [688]	77.1 [73.3–80.8]	15.9 [402]	21.5 [19.6–23.4]	12.5 [317]	25.6 [23.3–27.8]
χ^2^ *p*-Value	<0.001		<0.001		<0.001		<0.001	

Duration of travel = minutes for total daily journey to and from work.

doi:10.1371/journal.pmed.1001459.t002

Rural dwellers spent longer travelling to and from work than urban dwellers across all modes of travel. The mean daily travel time (to and from work) for those who walked to work was 39 min in rural dwellers and 22 min in urban dwellers. The mean daily travel time for those who bicycled to work was 48 min in rural dwellers and 26 min in urban dwellers. Mode of travel to work by respondent characteristics, which were treated as covariates in our analysis (age, sex, occupation, caste, standard of living, factory location, leisure time physical activity, daily fat intake, smoking status, alcohol use), are presented in Table 3.

**Table 3:** Mode of travel to work by respondent characteristics.

Characteristics	Private Transport	Public Transport	Walking	Bicycling	Total *n*	χ^2^ *p*-Value
	*n* [%]	*n* [%]	*n* [%]	*n* [%]		
**Age (y)**						
<35	216 [21.6]	305 [30.5]	143 [14.3]	336 [33.6]	1,000	<0.001
35–55	968 [38.3]	436 [17.3]	276 [10.9]	847 [33.5]	2,527	
55+	116 [30.9]	47 [12.5]	60 [16.0]	152 [40.5]	375	
**Sex**						
Female	1199 [35.2]	605 [17.8]	475 [14.0]	1,123 [33.0]	3,402	<0.001
Male	101 [20.2]	173 [36.6]	4 [0.8]	212 [42.4]	500	
**Occupation**						
Manual jobs	546 [23.3]	456 [19.5]	373 [15.9]	967 [41.3]	2,342	<0.001
Non-manual jobs	754 [48.3]	332 [21.3]	106 [6.8]	368 [23.6]	1,560	
**Caste/tribe status**						
Scheduled castes	176 [27.7]	125 [19.7]	99 [15.6]	236 [37.1]	636	<0.001
Scheduled tribes	38 [20.0]	25 [13.2]	42 [22.1]	85 [44.7]	190	
Other backward class	358 [27.9]	296 [23.1]	199 [15.5]	430 [33.5]	1,283	
General	728 [40.7]	341 [19.0]	138 [7.7]	584 [32.6]	1,791	
**Standard of living**						
Low	95 [6.9]	227 [16.5]	217 [15.8]	838 [60.9]	1,377	<0.001
Medium	561 [39.1]	375 [26.1]	169 [11.8]	330 [23.0]	1,435	
High	644 [59.1]	186 [17.1]	93 [8.5]	167 [15.3]	1,090	
**Smoking status**						
Non smoker	1,097 [33.6]	721 [22.1]	394 [12.1]	1,053 [32.3]	3,265	<0.001
Current smoker	203 [31.9]	67 [10.5]	85 [13.3]	282 [44.3]	637	
**Current alcohol intake**						
No	968 [32.1]	670 [22.2]	326 [10.8]	1,055 [35.0]	3,019	<0.001
Yes	332 [37.6]	118 [13.4]	153 [17.3]	280 [31.7]	883	
**Dietary fat intake**						
Low	346 [26.6]	194 [14.9]	597 [45.9]	164 [12.6]	1,301	<0.001
Medium	484 [37.2]	254 [19.5]	401 [30.8]	162 [12.5]	1,301	
High	470 [36.2]	340 [26.2]	337 [25.9]	153 [11.8]	1,300	
**Leisure time physical activity** a						
No	612 [30.2]	310 [15.3]	248 [12.3]	855 [42.2]	2,025	<0.001
Yes	688 [36.7]	478 [25.5]	231 [12.3]	480 [25.6]	1,877	
**Factory location**						
North India (Lucknow)	561 [45.6]	116 [9.4]	121 [9.8]	436 [35.3]	1,234	<0.001
Central India (Nagpur)	149 [18.6]	286 [35.7]	153 [19.1]	213 [26.6]	801	
South Central India (Hyderabad)	364 [36.3]	111 [11.1]	185 [18.4]	344 [34.3]	1,004	
South India (Bangalore)	226 [26.2]	275 [31.9]	20 [2.3]	342 [39.6]	863	
**TOTAL**	1,300 [33.3]	788 [20.2]	479 [12.3]	1335 [34.2]	3,902	

*p*-Values indicate whether sample characteristics differ significantly by mode of travel to work.

a Undertaking any leisure time physical activity in the past month.

doi:10.1371/journal.pmed.1001459.t003

### Mode of Travel to Work and Overweight and Obesity

**Figure 1:**
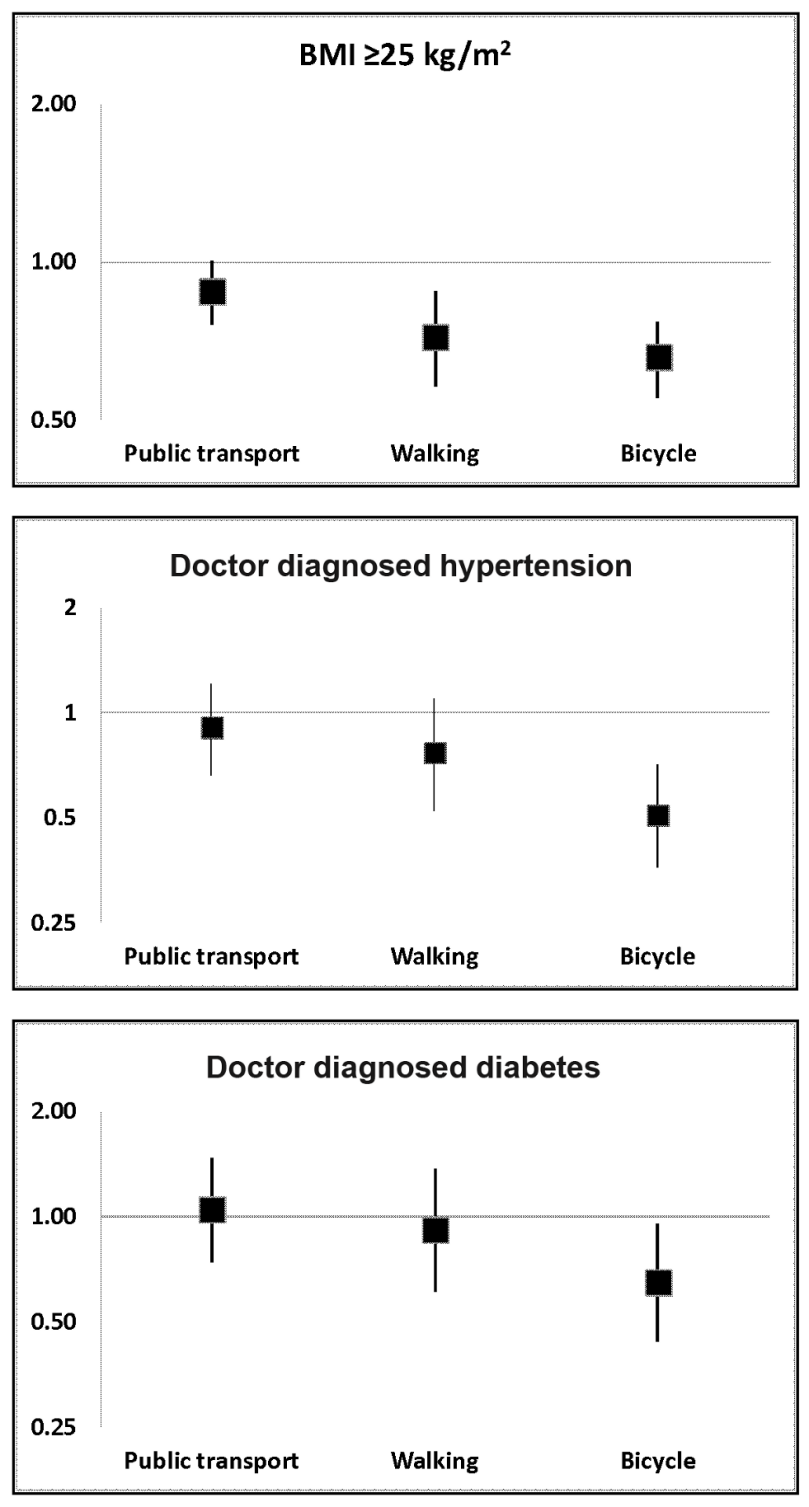
Adjusted risk ratios for mode of travel to work and overweight/obesity, hypertension, and diabetes. Note: Reference group is respondents who travel to work by private car. Risk ratios are adjusted for age, sex, caste, standard of living, occupation, factory location, leisure time physical activity, fat intake, smoking status, alcohol intake with an individual-specific random effect of sib-pair.

The prevalence of overweight or obesity was 50.0%, 37.6%, 24.9%, 24.2% (*p*<0.001) in participants who travelled to work by private transport, public transport, walking and bicycling, respectively. Respondents who used any mode of active travel to work were significantly less likely to be overweight or obese than those who travelled by private vehicle in the unadjusted analysis (Table 4). In the adjusted analysis, associations were attenuated but there was strong evidence that respondents who walked to work (adjusted risk ratio [ARR] 0.72; 95% CI 0.58–0.88) and those that bicycled to work (ARR 0.66; 95% CI 0.55–0.77) were less likely to be overweight or obese and those who bicycled were less likely to be obese (ARR 0.66; 95% CI 0.43–0.99). See Figure 1.

**Table 4:** Unadjusted and adjusted risk ratios (with 95% CI) for mode of travel to work and overweight and obesity, hypertension, and diabetes.

Mode of Travel to Work	BMI≥25 kg/m^2^ (*n* = 1,388)			BMI≥30 kg/m^2^ (*n* = 252)		
	Percent	URR^a^ [95% CI]	ARR^b^ [95% CI]	Percent	URR^a^ [95% CI]	ARR^b^ [95% CI]
Private car	50.0	1.00 [reference]	1.00 [reference]	9.6	1.00 [reference]	1.00 [reference]
Public transport	37.6	0.70 [0.59–0.80]	0.88 [0.76–1.01]	7.1	0.70 [0.48–1.01]	0.74 [0.49–1.12]
Walking	24.9	0.42 [0.33–0.53]	0.72 [0.58–0.88]	3.6	0.33 [0.18–0.58]	0.76 [0.42–1.34]
Bicycle	24.2	0.36 [0.31–0.44]	0.66 [0.55–0.77]	4.0	0.37 [0.26–0.55]	0.66 [0.43–0.99]

	Doctor diagnosed hypertension (*n* = 457)			Undiagnosed hypertension^c^ (*n* = 376)		
Private car	17.7	1.00 [reference]	1.00 [reference]	10.9	1.00 [reference]	1.00 [reference]
Public transport	11.8	0.63 [0.48–0.82]	0.91 [0.66–1.21]	8.3	0.73 [0.52–1.02]	0.91 [0.70–1.16]
Walking	9.8	0.51 [0.35–0.72]	0.77 [0.52–1.10]	9.8	0.90 [0.62–1.29]	0.97 [0.73–1.28]
Bicycle	6.5	0.30 [0.21–0.40]	0.51 [0.36–0.71]	9.2	0.84 [0.64–1.10]	0.69 [0.54–0.86]

	Doctor diagnosed diabetes (*n* = 283)			Undiagnosed diabetes^d^ (*n* = 122)		
Private car	10.8	1.00 [reference]	1.00 [reference]	4.8	1.00 [reference]	1.00 [reference]
Public transport	7.4	0.67 [0.48–0.92]	1.05 [0.74–1.47]	3.0	0.62 [0.37–1.04]	0.88 [0.49–1.53]
Walking	7.3	0.65 [0.45–0.96]	0.92 [0.61–1.37]	3.0	0.60 [0.32–1.12]	1.02 [0.51–1.98]
Bicycle	3.8	0.31 [0.22–0.96]	0.65 [0.44–0.95]	2.5	0.49 [0.31–0.79]	0.60 [0.34–1.04]

Percentages refer to the prevalence of each CVD risk factor by mode of travel to work.

a Unadjusted risk ratio.

b Adjusted risk ratio: adjusted for age, sex, caste, standard of living, occupation, factory location, leisure time physical activity, fat intake, smoking status, alcohol intake with an individual-specific random effect of sib-pair.

c Undiagnosed hypertension = SBP>140 mm Hg or DBP>90 mm Hg excluding those with doctor reported hypertension.

d Undiagnosed diabetes = fasting blood glucose ≥7 mmol/l excluding those with doctor reported diabetes.

doi:10.1371/journal.pmed.1001459.t004

### Mode of Travel to Work and Hypertension

The prevalence of diagnosed hypertension was 17.7%, 11.8%, 9.8%, 6.5% in participants who travelled to work by private transport, public transport, walking, and bicycling, respectively. Respondents who used any mode of active travel to work were less likely to have a doctor diagnosis of hypertension than those who travelled by private vehicle in the unadjusted analysis (Table 4). After adjustment all findings were attenuated, with only bicycling showing evidence of lower odds of hypertension (ARR 0.51; 95% CI 0.36–0.71) (see Figure 1). Bicycling to work was also associated with lower odds of having undiagnosed hypertension (ARR 0.69; 95% CI 0.54–0.86) in the adjusted analyses.

### Mode of Travel to Work and Diabetes

The prevalence of diagnosed diabetes was 10.8%, 7.4%, 7.3%, 3.8%, in participants who travelled to work by private transport, public transport, walking, and bicycling, respectively. Respondents who used any mode of active travel to work were significantly less likely to have a doctor diagnosis of diabetes than those who travelled by private vehicle in the unadjusted analysis (Table 4). Findings were markedly attenuated by adjustment with only bicycling associated with diabetes in the adjusted analysis (ARR 0.65; 95% CI 0.44–0.95) (see Figure 1). Respondents who bicycled to work were less likely to have undiagnosed diabetes in the unadjusted analysis but this finding attenuated in the adjusted analysis (ARR 0.60, 95% CI 0.34–1.04). After excluding respondents with diagnosed and undiagnosed diabetes, individuals who bicycled or walked to work were significantly less likely to have high HOMA values than those that used private transport in the unadjusted analysis, although this finding only remained significantly for bicycling in the adjusted analysis (Table 5).

**Table 5:** Unadjusted and adjusted risk ratios for mode of travel and duration of active travel with HOMA values.

Mode of Travel	HOMA Score (Top Third Versus Rest)			
	Percent Low HOMA	Percent High HOMA	URR^a^ [95% CI]	ARR^b^ [95% CI]
Private car	59.7	40.4	1.00 [reference]	1.00 [reference]
Public transport	63.1	36.9	0.90 [0.77–1.04]	1.00 [0.86–1.16]
Walking	75.5	24.5	0.57 [0.44–0.71]	0.88 [0.75–1.02]
Bicycle	72.1	27.9	0.64 [0.54–0.74]	0.81 [0.65–0.99]
**Duration of active travel**				
Walking				
No active travel	59.7	40.4	1.00 [reference]	1.00 [reference]
0 to 29 min/d	73.6	26.4	0.60 [0.43–0.78]	0.80 [0.60–1.01]
≥30 min/d	80.3	19.7	0.43 [0.27–0.68]	0.62 [0.38–0.94]
Bicycle				
No active travel	59.7	40.4	1.00 [reference]	1.00 [reference]
0 to 29 min/d	71.1	28.9	0.64 [0.53–0.77]	0.90 [0.74–1.07]
≥30 min/d	74.3	25.7	0.54 [0.41–0.71]	0.91 [0.69–1.15]

Percentages refer to the prevalence of low and high HOMA values by mode of travel to work. Duration of travel = minutes for total daily journey to and from work. HOMA score excludes doctor diagnosed diabetes cases and cases where fasting blood glucose ≥7 mmol/l.

a Unadjusted risk ratio.

b Adjusted risk ratio: adjusted for age, sex, caste, SLI, occupation, factory location, leisure time physical activity, fat intake, smoking status, alcohol intake with an individual-specific random effect of sib-pair.

doi:10.1371/journal.pmed.1001459.t005

### Duration of Bicycling and Walking to Work and Cardiovascular Risk Factors

Duration of bicycling to and from work showed a dose response relationship with CVD risk factors in both unadjusted and adjusted analyses. Compared with those who did not travel actively, those spent ≥30 min bicycling were less likely to be overweight (ARR 0.44; 95% CI 0.29–0.61) as were those who spent <30 min bicycling to work (ARR 0.73; 95% CI 0.61–0.88). Bicycling also showed similar dose response relationships with doctor diagnosed hypertension and with diagnosed diabetes (Findings are shown in Table 6). Duration of walking to work did not show a dose response pattern but both <30 and ≥30 min walking times were associated with reduced likelihood of overweight. Findings for doctor diagnosed hypertension showed only weak evidence of reduced odds ratios in adjusted analyses and no evidence of association for diagnosed diabetes. We ran sensitivity analyses to examine the impact of shorter (≥20 min) and longer (≥40 min) duration of daily active travel on risk factors (Tables S1 and S2). We found a similar prevalence (and associated adjusted odds ratios) of overweight, hypertension and diabetes when we set the cut point at ≥40 min active travel per day but higher prevalence of these risk factors when we set the cut point at ≥20 min per day. These findings suggest that the optimal duration of daily active travel for cardiovascular health in this sample is 30 min, which is consistent with international guidance [1].

**Table 6:** Unadjusted and adjusted risk ratios for duration of bicycling and walking to work and overweight/obesity, hypertension, and diabetes.

Duration of Active Transport	BMI≥25 kg/m^2^ (*n* = 1,388)			Doctor Diagnosed Hypertension (*n* = 457)			Doctor Diagnosed Diabetes (*n* = 283)		
	Percent	URR^a^	ARR^b^	Percent	URR^a^	ARR^b^	Percent	URR^a^	ARR^b^
**Walking**									
No active travel	50.0	1.00 [reference]	1.00 [reference]	17.7	1.00 [reference]	1.00 [reference]	10.8	1.00 [reference]	1.00 [reference]
0 to 29 min/d	25.4	0.40 [0.28–0.54]	0.56 [0.41–0.75]	10.7	0.57 [0.38–0.83]	0.79 [0.52–1.18]	8.1	0.75 [0.51–1.10]	1.00 [0.65–1.53]
≥30 min/d	23.5	0.35 [0.20–0.54]	0.66 [0.43–0.95]	7.6	0.37 [0.19–0.74]	0.68 [0.32–1.36]	5.3	0.49 [023–1.01]	0.91 [0.40–1.90]
**Bicycle**									
No active travel	50.0	1.00 [reference]	1.00 [reference]	17.7	1.00 [reference]	1.00 [reference]	10.8	1.00 [reference]	1.00 [reference]
0 to 29 min/d	27.6	0.44 [0.36–0.54]	0.73 [0.61–0.88]	7.6	0.36 [0.27–0.51]	0.54 [0.36–0.78]	4.1	0.38 [0.26–0.54]	0.66 [0.44–0.98]
≥30 min/d	15.9	0.21 [0.15–0.31]	0.44 [0.29–0.61]	3.9	0.18 [0.10–0.31]	0.28 [0.14–0.54]	2.8	0.26 [0.13–0.48]	0.48 [0.23–0.96]

Duration of travel = minutes for total daily journey to and from work.

a Unadjusted risk ratio.

b Adjusted risk ratio: adjusted for age, sex, caste, SLI, occupation, factory location, leisure time physical activity, fat intake, smoking status, alcohol intake with an individual-specific random effect of sib-pair.

doi:10.1371/journal.pmed.1001459.t006

In analyses examining between- and within-sib-pairs, the inverse associations between active modes of transport and overweight (BMI>25 kg/m^2^), hypertension and diabetes were similar in between-sibling-pairs and within-sibling-pairs, with no statistical evidence of differences between the effects (Table 7). The only exception to this is the association between walking and overweight, which is significant in the between-sibling-pairs analysis but not so (attenuates to null) in the within-sibling-pairs analysis. Sib-pair differences in the covariates used in the analysis are presented in Table S3. Our findings were substantially unchanged when we included BMI and METS (h/day) as additional covariates in our models (Table S4). We present associations between active travel and cardiovascular risk factors stratified by urban and rural respondents in Table S5. However, the number of rural respondents travelling to work by modes other than cycling was too small to meaningfully examine these associations in this group.

**Table 7:** Within- and between-pair effects of mode of travel to work and overweight/obesity, hypertension, and diabetes.

Outcome Measures	URR [95% CI]^a^	*p*-Value	ARR [95% CI]^b^	*p*-Value
**Overweight and obesity**				
Private car within sibs	1.39 [1.29–1.48]	<0.001	1.18 [1.06–1.30]	0.004
Private car between sibs	1.46 [1.38–1.52]	<0.001	1.21 [1.11–1.30]	<0.001
Test within = between	0.27		0.71	
Public transport within sibs	1.18 [1.01–1.35]	0.04	1.04 [0.86–1.24]	0.68
Public transport between sibs	1.02 [0.89–1.15]	0.75	1.07 [0.91–1.23]	0.42
Test within = between	0.16		0.84	
Walking within sibs	1.22 [0.95–1.53]	0.13	1.16 [0.87–1.51]	0.29
Walking between sibs	0.45 [0.34–1.58]	<0.001	0.67 [0.51–0.87]	0.002
Test within = between	<0.001		0.006	
Bicycle within sibs	0.39 [0.33–0.48]	<0.001	0.65 [0.53–0.81]	<0.001
Bicycle between sibs	0.59 [0.50–0.69]	<0.001	0.76 [0.65–0.91]	0.002
Test within = between	0.002		0.24	
**Doctor diagnosed hypertension**				
Private car within sibs	1.79 [1.42–2.22]	<0.001	1.39 [0.99–1.88]	0.06
Private car between sibs	1.88 [1.58–2.21]	<0.001	1.37 [1.05–1.75]	0.02
Test within = between	0.73		0.95	
Public transport within sibs	1.46 [1.03–2.01]	0.04	1.33 [0.84–2.03]	0.22
Public transport between sibs	0.85 [0.63–1.12]	0.25	1.05 [0.72–1.52]	0.78
Test within = between	0.02		0.44	
Walking within sibs	1.36 [0.84–2.12]	0.21	1.16 [0.63–2.04]	0.62
Walking between sibs	0.64 [0.44–0.95]	0.03	0.82 [0.50–1.33]	0.43
Test within = between	0.02		0.39	
Bicycle within sibs	0.29 [0.21–0.42]	<0.001	0.45 [0.28–0.69]	<0.001
Bicycle between sibs	0.52 [0.39–0.68]	<0.001	0.59 [0.41–0.86]	0.006
Test within = between	0.008		0.28	
**Doctor diagnosed diabetes**				
Private car within sibs	2.13 [1.51–2.91]	<0.001	1.28 [0.83–1.91]	0.27
Private car between sibs	1.75 [1.36–2.22]	<0.001	1.09 [0.79–1.47]	0.60
Test within = between	0.36		0.55	
Public transport within sibs	1.38 [0.84–2.19]	0.20	1.20 [0.69–2.04]	0.52
Public transport between sibs	0.89 [0.62–1.26]	0.51	1.23 [0.80–1.84]	0.33
Test within = between	0.16		0.95	
Walking within sibs	1.39 [0.75–2.47]	0.29	1.06 [0.53–2.05]	0.85
Walking between sibs	0.87 [0.56–1.33]	0.51	1.00 [0.59–1.65]	0.99
Test within = between	0.23		0.88	
Bicycle within sibs	0.26 [0.17–0.40]	<0.001	0.57 [0.34–0.95]	0.03
Bicycle between sibs	0.50 [0.35–0.71]	<0.001	0.67 [0.44–1.02]	0.06
Test within = between	0.01		0.59	

a Unadjusted relative risk ratio.

b Adjusted risk ratio: adjusted for age, sex, caste/tribe status, standard of living, occupation, leisure time physical activity, fat intake, smoking status, alcohol intake, and factory location.

doi:10.1371/journal.pmed.1001459.t007

## Discussion

We found a lower likelihood of overweight in participants that walked or bicycled to work compared to those that used private transport. Participants who bicycled to work were less likely to have hypertension or diabetes than those that travelled to work by private transport. These findings are consistent with a growing evidence base derived from studies conducted in high-income settings and upper middle-income countries [23]. This includes evidence that individuals who take active transport to work have higher overall levels of physical activity and are less likely to be overweight or obese [24]–[26]. A meta-analysis of studies conducted prior to 2007 found that active travel to work was associated with an 11% reduction in cardiovascular outcomes (pooling cardiovascular mortality, incident coronary heart disease, stroke, hypertension diabetes) [7]. The CARDIA study found that active commuting was inversely associated with BMI, obesity, triglyceride level, blood pressure, and fasting insulin and positively associated with high density lipoprotein (HDL) cholesterol [27]. An earlier study conducted in Copenhagen found that bicycling to work was associated with 28% decrease in mortality after adjustment for leisure time physical activity [28]. A Finnish study found that daily walking or cycling to and from work for more than 30 min was inversely associated with risk of type 2 diabetes [29].

We were able to identify a dose response relationship between duration of bicycling to work and being overweight, having hypertension and diabetes in both unadjusted and adjusted analyses, which strengthens a causal interpretation of these associations. For duration of walking, dose response relationships were seen in unadjusted analyses but attenuated for hypertension and diabetes. The similar findings in our main analyses, which allow for the sib-pair design, to those from our analyses that examine associations in between and within sib-pairs, provide stronger evidence that these associations are robust. The only exception to this is the association between walking to work and overweight, which is significant in the between-sibling-pairs analysis but not so (attenuates to null) in the within-sibling-pairs analysis. While this does raise a question about the robustness of this association, the most likely explanation is that this is a chance finding given the consistency of other comparisons made. The strengths of our study include the large sample and use of sibling pair design. However, like all cross-sectional studies, the data from this study may be prone to recall bias and there remains the possibility of residual confounding (the sib pair design increases the potential for confounding although it reduces the potential for confounding by genetic factors). For example, duration of active travel is based on self-report and may not be accurate. As only the main mode of travel was recorded, we did not have information on how long respondents spent walking (or cycling) to access points if public transport was used. In an effort to address the possibility of reverse causality, i.e., individuals with diagnosed hypertension may receive physician advice to increase physical activity, we examined associations between active travel and undiagnosed hypertension and diabetes. Several of these findings were consistent with a protective effect of active travel although only the association between bicycling and undiagnosed hypertension was statistically significant. Longitudinal studies are required to better determine cause and effect. There is a risk of selection bias in this study due to non-response and missing values. Our sample size was too small to examine associations between mode of active travel and cardiovascular risk factors in rural and urban groups separately. We did not have information on exposure to ambient or household air pollution, which has been associated with an elevated risk of hypertension and diabetes [30],[31]. However, as this exposure is likely to be socially patterned, adjustment for socio-economic factors (which is done in our analyses) will provide some control for these possible effects. Our urban sample consisted of factory employees whose patterns of active travel may differ from urban residents generally. Mean travel times for walking (22 min) and cycling (26 min) to and from work among respondents living in urban areas appear low indicating that many lived close to the factories. This may mean that the proportion of people undertaking active travel in our sample is higher and the associated cardiovascular benefits identified here are lower (given the mean duration is lower than that recommend by international guidance [1]) than is typical for similar populations in India. Unfortunately there are few data on patterns of active travel in India available to compare our findings with. However, the high percentage of urban respondents using private transport for commuting (45%) reflects recent dramatic growth in car and motorbike ownership and lack of investment in public transport infrastructure in India [5],[6]. We were not able to examine non-cardiovascular outcomes that may be of interest (e.g., injuries sustained during active travel, exposure to air pollution, effects on mental health outcomes).

India is facing a growing burden of NCDs, particularly from diabetes and CVD [11],[32]. For example, total deaths from CVD are projected to increase from 2.7 million in 2004 to 4.0 million in 2030 33]. While the prevalence of overweight and obesity remained relatively stable in India since the 1990s [34],[35], this is projected to increase rapidly in the next two decades [36]. Our findings indicate that increasing active travel could be important in restraining this increase. Differences in active travel in urban and rural groups found in our study may partly explain the higher prevalence of diabetes and hypertension in urban India and the negative impact of rural-to-urban migration on CVD risk factors [10],[37].

Efforts to increase active travel in urban areas and halt declines in rural areas should be integral to strategies to maintain healthy weight and prevent NCDs in India. This should include greater investment in public transport and improving the safety and convenience of bicycling and walking in Indian towns and cities [38]–[40]. Specific measures to discourage car use should also be considered and could include carbon rationing, road pricing, car parking restrictions, and reduced speed limits [41]. Direct to consumer subsidies and workplace facilities, such as bicycle parking and showers, to encourage active travel over private vehicle use have been associated with improved health outcomes in high-income countries and should be considered [42]. Further research evaluating the impact of interventions to increase active travel in India and other LMICs is warranted. An assessment of potential future health benefits of increasing active travel should account for the potentially deleterious effects of increased exposure to air pollution and road traffic injuries alongside the substantial long term benefits of reduced carbon emissions.

## Supporting Information

Table S1 **Unadjusted and adjusted risk ratios for duration of bicycling and walking to work and overweight/obesity, hypertension, and diabetes (20-min cutoff point).**
(DOCX)

Table S2 **Unadjusted and adjusted risk ratios for duration of bicycling and walking to work and overweight/obesity, hypertension, and diabetes (40-min cutoff point).**
(DOCX)

Table S3 **Sib pair differences in characteristics.**
(DOCX)

Table S4 **Unadjusted and adjusted risk ratios for mode of travel to work and overweight and obesity, Hypertension, and diabetes (sensitivity analysis adjusting for BMI and total METS).**
(DOCX)

Table S5 **Unadjusted and adjusted risk ratios for mode of travel to work and overweight and obesity, hypertension and diabetes stratified by area of residence.**
(DOCX)

## Abbreviations

AAR: adjusted risk ratio
BMI: body mass index
BP: blood pressure
HOMA: homeostasis model assessment
IMS: Indian Migration Study
LMICs: low- and middle-income countries
MET: metabolic equivalent task
NCD: noncommunicable disease
SD: standard deviation
SLI: standard of living index
WHO: World Health Organization
