# Supplementary material for: Associations between Active Travel to Work and Overweight, Hypertension, and Diabetes in India: A Cross-Sectional Study
Source: PLoS Med. 2013 Jun 11;10(6):e1001459. doi: 10.1371/journal.pmed.1001459 (PMC3679004; doi:10.1371/journal.pmed.1001459)
Supplement: Table S1 — Unadjusted and adjusted risk ratios for duration of bicycling and walking to work and overweight/obesity, hypertension, and diabetes (20-min cutoff point). (DOCX) [file pmed.1001459.s001.docx]

**Table S1: Unadjusted and adjusted risk ratios for duration of bicycling and walking to work and overweight /obesity, hypertension and diabetes (20 minutes cutoff point)**

| **Duration of active transport** | **BMI ≥25 kg/m^2^ (n=1388)** | | | **Doctor diagnosedhypertension (n=457)** | | | **Doctor diagnosed diabetes (n=283)** | | |
| --- | --- | --- | --- | --- | --- | --- | --- | --- | --- |
|  | **%** | **URR^a^[95%CI]** | **ARR^b^[95%CI]** | **%** | **URR^a^[95%CI]** | **ARR^b^[95%CI]** | **%** | **URR^a^[95%CI]** | **ARR^b^[95%CI]** |
| **Walking** |  |  |  |  |  |  |  |  |  |
| No active travel | 50.0 | 1.00[reference] | 1.00[reference] | 17.7 | 1.00[reference] | 1.00[reference] | 10.8 | 1.00[reference] | 1.00[reference] |
| 0 to 19 mins/day | 22.9 | 0.35[0.21-0.55] | 0.47[0.29-0.71 | 11.4 | 0.62[0.35-1.03] | 0.85[0.46-1.45] | 7.8 | 0.72[0.40-1.28] | 0.97[0.51-1.77] |
| ≥20 mins/day | 25.7 | 0.39[0.28-0.54] | 0.65[0.48-0.84] | 9.2 | 0.48[0.31-0.72] | 0.73[0.45-1.13] | 7.1 | 0.66[0.43-0.99] | 0.99[0.62-1.54] |
| **Bicycle** |  |  |  |  |  |  |  |  |  |
| No active travel | 50.0 | 1.00[reference] | 1.00[reference] | 17.7 | 1.00[reference] | 1.00[reference] | 10.8 | 1.00[reference] | 1.00[reference] |
| 0 to 19 mins/day | 31.2 | 0.52[0.41-0.65] | 0.74[0.59-0.90] | 9.4 | 0.47[0.32-0.67] | 0.60[0.38-0.89] | 5.7 | 0.53[0.34-0.79] | 0.77[0.49-1.19] |
| ≥20 mins/day | 20.6 | 0.31[0.23-0.39] | 0.62[0.48-0.77] | 5.0 | 0.23[0.15-0.35] | 0.37[0.23-0.60] | 2.7 | 0.25[0.15-0.39] | 0.48[0.28-0.80] |

Note: Duration of walking and cycling = minutes for total daily journey to and from work

^a^ Unadjusted risk ratio

^b^ Adjusted risk ratio: adjusted for age, sex, caste, standard of living index, occupation, factory location, current smoking, current alcohol intake, fat intake, leisuretime physical activity, with an individual-specific random effect of sib-pair
