# Supplementary material for: Associations between Active Travel to Work and Overweight, Hypertension, and Diabetes in India: A Cross-Sectional Study
Source: PLoS Med. 2013 Jun 11;10(6):e1001459. doi: 10.1371/journal.pmed.1001459 (PMC3679004; doi:10.1371/journal.pmed.1001459)
Supplement: Table S2 — Unadjusted and adjusted risk ratios for duration of bicycling and walking to work and overweight/obesity, hypertension, and diabetes (40-min cutoff point). (DOCX) [file pmed.1001459.s002.docx]

**Table S2: Unadjusted and adjusted risk ratios for duration of bicycling and walking to work and overweight /obesity, hypertension and diabetes (40 minutes cutoff point)**

| **Duration of active transport** | **BMI ≥25 kg/m^2^ (n=1388)** | | | **Doctor diagnosed hypertension (n=457)** | | | **Doctor diagnosed diabetes (n=283)** | | |
| --- | --- | --- | --- | --- | --- | --- | --- | --- | --- |
|  | **%** | **URR^a^[95%CI]** | **ARR^b^[95%CI]** | **%** | **URR^a^[95%CI]** | **ARR^b^[95%CI]** | **%** | **URR^a^[95%CI]** | **ARR^b^[95%CI]** |
| **Walking** |  |  |  |  |  |  |  |  |  |
| No active travel | 50.0 | 1.00[reference] | 1.00[reference] | 17.7 | 1.00[reference] | 1.00[reference] | 10.8 | 1.00[reference] | 1.00[reference] |
| 0 to 39 mins/day | 25.5 | 0.40[0.29-0.54] | 0.56[0.41-0.74] | 10.8 | 0.58[0.40-0.84] | 0.79[0.52-1.18] | 8.2 | 0.76[0.51-1.11] | 1.00[0.65-1.53] |
| ≥40 mins/day | 23.4 | 0.33[0.20-0.54] | 0.68[0.44-0.96] | 7.3 | 0.36[0.18-0.71] | 0.68[0.31-1.36] | 5.1 | 0.48[0.22-0.97] | 0.91[0.41-1.89] |
| **Bicycle** |  |  |  |  |  |  |  |  |  |
| No active travel | 50.0 | 1.00[reference] | 1.00[reference] | 17.7 | 1.00[reference] | 1.00[reference] | 10.8 | 1.00[reference] | 1.00[reference] |
| 0 to 39 mins/day | 27.9 | 0.45[0.36-0.55] | 0.74[0.61-0.88] | 7.7 | 0.37[0.27-0.51] | 0.54[0.36-0.78] | 4.2 | 0.38[0.26-0.55] | 0.66[0.44-0.98] |
| ≥40 mins/day | 15.6 | 0.20[0.13-0.29] | 0.43[0.29-0.59] | 3.8 | 0.17[0.10-0.31] | 0.28[0.13-0.54] | 2.8 | 0.25[0.13-0.47] | 0.48[0.23-0.96] |

Note: Duration of walking and cycling = minutes for total daily journey to and from work

^a^ Unadjusted risk ratio

^b^ Adjusted risk ratio: adjusted for age, sex, caste, standard of living index, occupation, factory location, current smoking, current alcohol intake, fat intake, leisuretime physical activity, with an individual-specific random effect of sib-pair
