# Supplementary material for: Associations between Active Travel to Work and Overweight, Hypertension, and Diabetes in India: A Cross-Sectional Study
Source: PLoS Med. 2013 Jun 11;10(6):e1001459. doi: 10.1371/journal.pmed.1001459 (PMC3679004; doi:10.1371/journal.pmed.1001459)
Supplement: Table S3 — Sib pair differences in characteristics. (DOCX) [file pmed.1001459.s003.docx]

**Table S3: Sib pair differences in characteristics**

| **Characteristics** | **Sibpairs** | **Migrant (urban-rural)** | **p value** | **Nonmigrant (urban-urban)** | **p value** |
| --- | --- | --- | --- | --- | --- |
| Mean age (years) | Sibling | 40.3 |  | 40.2 |  |
|  | Index | 44.7 |  | 43.7 |  |
| % Men | Sibling | 90.0 | 0.01 | 81.7 | 0.04 |
|  | Index | 93.1 |  | 77.3 |  |
| **Occupation** |  |  |  |  |  |
| Manual jobs | Sibling | 75.1 | <0.001 | 44.8 | 0.97 |
|  | Index | 60.2 |  | 44.9 |  |
| Non-manual jobs | Sibling | 25.1 |  | 55.2 |  |
|  | Index | 40.4 |  | 55.1 |  |
| **Standard of living** |  |  |  |  |  |
| Low | Sibling | 69.8 | <0.001 | 24.3 | <0.001 |
|  | Index | 15.4 |  | 11.5 |  |
| Medium | Sibling | 22.5 |  | 41.8 |  |
|  | Index | 45.7 |  | 47.0 |  |
| High | Sibling | 8.7 |  | 33.9 |  |
|  | Index | 39.0 |  | 41.5 |  |
| **Current smoker** | Sibling | 19.5 | 0.005 | 15.3 | 0.20 |
|  | Index | 15.2 |  | 12.9 |  |
| **Current alcohol intake** | Sibling | 22.5 | 0.02 | 20.5 | 0.26 |
|  | Index | 26.5 |  | 18.1 |  |
| **Mean leisure time PA*** | Sibling | 1.10[±2.20] |  | 1.50[±2.38] |  |
|  | Index | 1.64[±2.14] |  | 1.68[±2.28] |  |
| **Mean dietary fat intake**** | Sibling | 77.3[±37.2] |  | 94.6[±36.8] |  |
|  | Index | 92.5[±38.2] |  | 94.1[±34.9] |  |
| **BMI ≥25 kg/m^2^** | Sibling | 19.1 | <0.001 | 42.9 | 0.005 |
|  | Index | 41.9 |  | 50.6 |  |
| **BMI ≥30 kg/m^2^** | Sibling | 2.9 | 0.01 | 11.0 | 0.58 |
|  | Index | 4.9 |  | 12.0 |  |
| **Doctor diagnosed hypertension** | Sibling | 5.1 | <0.001 | 9.4 | <0.001 |
|  | Index | 15.7 |  | 20.5 |  |
| **Undiagosed hypertension** | Sibling | 8.4 | 0.08 | 10.3 | 0.89 |
|  | Index | 10.5 |  | 10.0 |  |
| **Doctor diagnosed diabetes** | Sibling | 2.8 | <0.001 | 7.4 | 0.03 |
|  | Index | 10.2 |  | 10.9 |  |
| **Undiagnosed diabetes** | Sibling | 2.3 | 0.04 | 4.2 | 0.98 |
|  | Index | 3.8 |  | 4.2 |  |
| **High HOMA score***** | Sibling | 25.7 | <0.001 | 41.4 | 0.45 |
|  | Index | 34.7 |  | 39.2 |  |

Differences are expressed as percentages unless stated as means. p values indicate whether sample characteristics differ significantly in factory workers (index) and their siblings

*Mean leisure time physical activity (MET hours per day)

**Mean dietary fat intake (grams per day)

***HOMA score excludes doctor diagnosed diabetes cases and cases where fasting blood glucose >=7mmol/l
