# Supplementary material for: Associations between Active Travel to Work and Overweight, Hypertension, and Diabetes in India: A Cross-Sectional Study
Source: PLoS Med. 2013 Jun 11;10(6):e1001459. doi: 10.1371/journal.pmed.1001459 (PMC3679004; doi:10.1371/journal.pmed.1001459)
Supplement: Table S4 — Unadjusted and adjusted risk ratios for mode of travel to work and overweight and obesity, Hypertension, and diabetes (sensitivity analysis adjusting for BMI and total METS). (DOCX) [file pmed.1001459.s004.docx]

**Table S4: Unadjusted and adjusted risk ratios for mode of travel to work and overweight and obesity,**

**hypertension and diabetes (sensitivity analysis adjusting for BMI and total METS)**

| **Mode of transport to work** | **BMI≥25 kg/m^2^**  **(n=1388)** | | | **BMI≥30 kg/m^2^**  **(n=252)** | | |
| --- | --- | --- | --- | --- | --- | --- |
|  | **%** | **URR^a^[95%CI]** | **ARR^b^[95%CI]** | **%** | **URR^a^[95%CI]** | **ARR^b^[95%CI]** |
| Private car | 50.0 | 1.00[reference] | 1.00[reference] | 9.6 | 1.00[reference] | 1.00[reference] |
| Public transport | 37.6 | 0.70[0.59-0.80] | 0.88[0.76-1.01] | 7.1 | 0.70[0.48-1.01] | 0.74[0.49-1.12] |
| Walking | 24.9 | 0.42[0.33-0.53] | 0.72[0.58-0.88] | 3.6 | 0.33[0.18-0.58] | 0.76[0.42-1.34] |
| Bicycle | 24.2 | 0.36[0.31-0.44] | 0.66[0.55-0.77] | 4.0 | 0.37[0.26-0.55] | 0.66[0.43-0.99] |
|  | | | | | | |
|  | **Doctor diagnosed hypertension (n=457)** | | | **Undiagnosed hypertension* (n=376)** | | |
|  | **%** | **URR^a^[95%CI]** | **ARR^b^[95%CI]** | **%** | **URR^a^[95%CI]** | **ARR^b^[95%CI]** |
| Private car | 17.7 | 1.00[reference] | 1.00[reference] | 10.9 | 1.00[reference] | 1.00[reference] |
| Public transport | 11.8 | 0.63[0.48-0.82] | 0.92[0.78-1.13] | 8.3 | 0.73[0.52-1.02] | 0.95[0.83-1.11] |
| Walking | 9.8 | 0.51[0.35-0.72] | 0.91[0.69-1.12] | 9.8 | 0.90[0.62-1.29] | 1.06[0.90-1.21] |
| Bicycle | 6.5 | 0.30[0.21-0.40] | 0.73[0.57-0.91] | 9.2 | 0.84[0.64-1.10] | 0.91[0.78-1.04] |
|  |  |  |  |  |  |  |
|  | **Doctor diagnosed diabetes (n=283)** | | | **Undiagnosed diabetes# (n=122)** | | |
|  | **%** | **URR^a^[95%CI]** | **ARR^b^[95%CI]** | **%** | **URR^a^[95%CI]** | **ARR^b^[95%CI]** |
| Private car | 10.8 | 1.00[reference] | 1.00[reference] | 4.8 | 1.00[reference] | 1.00[reference] |
| Public transport | 7.4 | 0.67[0.48-0.92] | 1.08[0.76-1.51] | 3.0 | 0.62[0.37-1.04] | 0.84[0.46-1.50] |
| Walking | 7.3 | 0.65[0.45-0.96] | 0.89[0.59-1.34] | 3.0 | 0.60[0.32-1.12] | 1.32[0.64-2.59] |
| Bicycle | 3.8 | 0.31[0.22-0.96] | 0.69[0.46-1.00] | 2.5 | 0.49[0.31-0.79] | 0.83[0.46-1.44] |
|  |  |  |  |  |  |  |

^a^ Unadjusted risk ratios

^b^ Adjusted risk ratios; adjusted for age, sex, caste, standard of living, occupation, factory location, smoking, current alcohol intake, fat intake, total METS, leisuretime physical activity, BMI with an individual-specific random effect of sib-pair

*Undiagnosed hypertension = SBP > 140 mm Hg and DBP > 90 mm Hg excluding those with doctor reported hypertension

# Undiagnosed diabetes = fasting blood glucose >=7mmol/l excluding those with doctor reported diabetes
