# Supplementary material for: Associations between Active Travel to Work and Overweight, Hypertension, and Diabetes in India: A Cross-Sectional Study
Source: PLoS Med. 2013 Jun 11;10(6):e1001459. doi: 10.1371/journal.pmed.1001459 (PMC3679004; doi:10.1371/journal.pmed.1001459)
Supplement: Table S5 — Unadjusted and adjusted risk ratios for mode of travel to work and overweight and obesity, hypertension and diabetes stratified by area of residence. (DOCX) [file pmed.1001459.s005.docx]

**Table S5: Unadjusted and adjusted risk ratios for mode of travel to work and overweight and obesity, hypertension and diabetes stratified by area of residence**

| **Mode of transport** | **BMI ≥25 kg/m^2^ (n=1388)** | | | **Doctor diagnosed hypertension (n=457)** | | | **Doctor diagnosed diabetes (n=283)** | | |
| --- | --- | --- | --- | --- | --- | --- | --- | --- | --- |
|  | **%** | **URR^a^** | **ARR^b^** | **%** | **URR^a^** | **ARR^b^** | **%** | **URR^a^** | **ARR^b^** |
| **Urban** |  |  |  |  |  |  |  |  |  |
| Private car | 51.8 | 1.00[reference] | 1.00[reference] | 19.5 | 1.00[reference] | 1.00[reference] | 11.5 | 1.00[reference] | 1.00[reference] |
| Public transport | 39.1 | 0.69[0.58-0.82] | 0.92[0.79-1.05] | 11.9 | 0.55[0.41-0.75] | 0.86[0.61-1.18] | 7.6 | 0.64[0.45-0.89] | 1.03[0.37-2.63] |
| Walking | 30.1 | 0.49[0.37-0.63] | 0.68[0.53-0.85] | 12.3 | 0.55[0.37-0.83] | 0.73[0.46-1.10] | 10.4 | 0.88[0.59-1.31] | 0.21[0.04-0.93] |
| Bicycling | 44.3 | 0.80[0.66-0.94] | 0.91[0.76-1.06] | 11.7 | 0.53[0.36-0.76] | 0.66[0.44-0.97] | 7.5 | 0.62[0.40-0.94] | 0.37[0.17-0.78] |
| **Rural** |  |  |  |  |  |  |  |  |  |
| Private car | 38.0 | 1.00[reference] | 1.00[reference] | 5.9 | 1.00[reference] | 1.00[reference] | 5.9 | 1.00[reference] | 1.00[reference] |
| Public transport | 27.0 | 0.71[1.36e-18-2.60e+17] | 0.73[0.40-1.19] | 11.0 | 1.88[9.95e-10-4.03e+09] | 1.85[0.68-4.48] | 6.0 | 0.99[0.69-1.41] | 1.16[0.36-3.33] |
| Walking | 14.8 | 0.39[4.54e-39-1.68e+37] | 0.53[0.28-0.97] | 4.9 | 0.85[0.02-12.4] | 0.98[0.33-2.71] | 1.2 | 0.99[0.65-1.50] | 0.34[0.06-1.66] |
| Bicycling | 15.5 | 0.71[8.12e-38-1.06e+36] | 0.62[0.31-1.08] | 4.3 | 0.73[0.00-1.65] | 0.58[0.24-1.39] | 2.1 | 0.81[0.52-1.24] | 066[0.25-1.67] |

^a^ Unadjusted risk ratio

^b^ Adjusted risk ratio: adjusted for age, sex, caste, standard of living, occupation, factory location, smoking, current alcohol intake, fat intake, leisuretime physical activity, with an individual-specific random effect of sib-pair

*Undiagnosed hypertension = SBP > 140 mm Hg and DBP > 90 mm Hg excluding those with doctor reported hypertension

# Undiagnosed diabetes = fasting blood glucose >=7mmol/l excluding those with doctor reported diabetes
